# Cytoskeletal Remodeling Mimics Endothelial Response to Microgravity

**DOI:** 10.3389/fcell.2021.733573

**Published:** 2021-09-09

**Authors:** Laura Locatelli, Jeanette A. M. Maier

**Affiliations:** ^1^Department of Biomedical and Clinical Sciences L. Sacco, Università di Milano, Milan, Italy; ^2^Interdisciplinary Centre for Nanostructured Materials and Interfaces, Università di Milano, Milan, Italy

**Keywords:** human endothelial cells, microgravity, stress, TRPM7, cytoskeleton

## Abstract

Mechanical cues contribute to the maintenance of a healthy endothelium, which is essential for vascular integrity. Indeed endothelial cells are mechanosensors that integrate the forces in the form of biochemical signals. The cytoskeleton is fundamental in sensing mechanical stimuli and activating specific signaling pathways. Because the cytoskeleton is very rapidly remodeled in endothelial cells exposed to microgravity, we investigated whether the disruption of actin polymerization by cytochalasin D in 1g condition triggers and orchestrates responses similar to those occurring in micro- and macro-vascular endothelial cells upon gravitational unloading. We focused our attention on the effect of simulated microgravity on stress proteins and transient receptor potential melastatin 7 (TRPM7), a cation channel that acts as a mechanosensor and modulates endothelial cell proliferation and stress response. Simulated microgravity downregulates TRPM7 in both cell types. However, 24 h of treatment with cytochalasin D decreases the amounts of TRPM7 only in macrovascular endothelial cells, suggesting that the regulation and the role of TRPM7 in microvascular cells are more complex than expected. The 24 h culture in the presence of cytochalasin D mimics the effect of simulated microgravity in modulating stress response in micro- and macro-vascular endothelial cells. We conclude that cytoskeletal disruption might mediate some effects of microgravity in endothelial cells.

## Introduction

At the interface between the blood and the tissues, vascular endothelial cells (EC) are constantly exposed to a myriad of stimuli which finely shape their phenotype and tune their function. Indeed metabolites, hormones, growth factors, cytokines, a high concentration of oxygen, and also mechanical stresses induce a series of active adaptive processes to maintain cell homeostasis (Michiels, 2003; Schober, 2008; Deng et al., 2014; Zhang and Dong, 2014; Zhang and Li, 2017; Marsboom and Rehman, 2018). This is crucial because endothelial cells are in charge of the integrity of the whole vascular tree, which means, among others, to grant adequate perfusion and nourishment to all the tissues (Davies, 2009). Consequently, while a healthy endothelium is fundamental for mammalian survival, endothelial dysfunction is at the roots of many diseases (Drexler and Hornig, 1999; Vanhoutte et al., 2017; Sun et al., 2019; Morris et al., 2020; Siddiqi et al., 2021). EC sense and react to mechanical stimuli through complex pathways that translate mechanical forces into biochemical signals that ultimately carve cell phenotype and function (Gliemann et al., 2017). The endothelium is exposed simultaneously to three types of mechanical forces: shear stress, circumferential strain, and, although often forgotten, gravity. Frictional shear stress is generated by blood flow, acts parallel to the vessel wall, and promotes endothelial homeostasis and vascular health (Baeyens et al., 2016). Circumferential stress is due to blood pressure and influences endothelial morphology and gene expression (Ohashi et al., 2007). The impact of gravity on organisms has been overlooked until recently because this force has remained constant since the origin of our planet. As space exploration began, the relevance of gravity in modulating the function of our tissues and cells became clear. In astronauts, weightlessness alters endothelial performance, thus contributing to cardiovascular deconditioning and to an impairment of endothelium-dependent functions in the microcirculation (Coupé et al., 2009), which might be partly responsible for several microgravity-associated pathophysiological processes, among which is muscle and bone loss. Several *in vitro* studies have demonstrated how simulated or real microgravity and hypergravity forge endothelial behavior (Carlsson et al., 2003; Cotrupi et al., 2005; Versari et al., 2007, 2013; Kapitonova et al., 2012; Grenon et al., 2013; Maier et al., 2015; De Cesari et al., 2020). Alterations of cell proliferation, migration, signal transduction, and gene expression have been reported in macro- and micro-vascular EC (Carlsson et al., 2003; Cotrupi et al., 2005; Versari et al., 2007, 2013; Kapitonova et al., 2012; Grenon et al., 2013; Maier et al., 2015). However, the mechanisms involved in EC response to gravitational cues remain unclear. It is well known that the endothelium is equipped with machinery that perceives mechanical forces and converts them in biochemical signals (Chatterjee and Fisher, 2014). Channels, glycocalyx, integrins, cell receptors, and cytoskeleton, among others, are involved in mechanosensing and mechanosignaling to intracellular signaling pathways (Harding and Ebong, 2014; Yamashiro and Yanagisawa, 2020). The cytoskeleton is a dynamic structure fundamental not only for the maintenance of the shape and the inner organization of the cell but also for the correct function of different organelles and the transport of molecules within the cell (Martino et al., 2018). EC in microgravity, like all the eukaryotic cells studied until now, remodel their cytoskeleton (Carlsson et al., 2003; Versari et al., 2007; Kapitonova et al., 2012; Janmaleki et al., 2016), and this might be one of the mechanisms impacting on alterations of cell behavior (Carlsson et al., 2003; Cotrupi et al., 2005; Versari et al., 2007, 2013; Kapitonova et al., 2012; Grenon et al., 2013; Maier et al., 2015).

Recently, channels of the transient receptor potential family emerged as mechanosensors (Negri et al., 2019). In particular, transient receptor potential melastatin 7 (TRPM7), a ubiquitous cation channel with a cytosolic α-kinase domain, acts as a mechanosensor of hydraulic resistance in two different tumor cell lines and is essential in guiding cell migration (Zhu et al., 2019). In mesenchymal stem cells, TRPM7 is involved in the regulation of osteogenic differentiation in response to shear stress (Liu et al., 2015). In endothelial cells exposed to shear stress, TRPM7 is upregulated in comparison to the controls grown under static conditions (Thilo et al., 2012). In addition, it contributes to the regulation of endothelial migration and proliferation (Inoue and Xiong, 2009; Baldoli et al., 2013). To the best of our knowledge, no data are available at the moment about the modulation of TRPM7 in microgravity.

In this paper, we investigated the role of cytoskeletal disorganization in orchestrating endothelial response to simulated microgravity. Because of the heterogeneity of the endothelium, we used two different types of EC, i.e., human endothelial cells from umbilical vein (HUVEC) and human dermal microvascular endothelial cells (HDMEC), a model of macro- and microvascular EC, respectively, which have been used for studies in real and simulated microgravity (Shi et al., 2012; Grenon et al., 2013; Versari et al., 2013; Barravecchia et al., 2018; Cazzaniga et al., 2019; Locatelli et al., 2020).

## Materials and Methods

### Cell Culture

HUVEC and HDMEC were used as a model for macrovascular and microvascular EC, respectively. The HUVEC were from ATCC and serially passaged in M199 containing 10% of fetal bovine serum (FBS), endothelial cell growth factor (150 μg/ml), glutamine (2 mM), sodium pyruvate (1 mM), and heparin (5 U/ml) on 2% gelatin-coated dishes. The HDMEC were obtained from Lonza (Basel Switzerland) and grown in MCDB131 containing epidermal growth factor (10 ng/ml) and 10% FBS and glutamine (2 mM) on 2% gelatin-coated dishes. All culture reagents were from Gibco (Thermo Fisher Scientific, Waltham, MA, United States).

To simulate microgravity, we utilized the Rotating Wall Vessels (RWV) (Synthecom Inc, Houston, TX, United States) after seeding the cells on microcarrier beads (Cytodex 3, Sigma, St. Louis, MO, United States) (Carlsson et al., 2003). As controls in 1g condition, cells grown on beads were cultured in the vessels not undergoing rotation. Both samples were cultured at 37°C and 5% CO_2_. The experiments were performed after 4 and 10 days of exposure to simulated microgravity.

To mimic cytoskeletal disruption in 1g, we treated cells cultured in Petri dishes or in multiwell plates with cytochalasin D (CYT D) (Sigma Aldrich, St. Louis, MO, United States). CYT D is a toxin that binds actin and induces its depolymerization. We performed dose- and time-dependent experiments to test cell viability and cytoskeletal remodeling. On the bases of these experiments and considering CYT D cytotoxicity, we performed experiments on CYT D-treated HUVEC for 24 and 96 h and on CYT D-treated HDMEC for 24 and 72 h.

### MTT Assay and Cell Count

HUVEC and HDMEC were seeded in 96-well plates and exposed 24 h later to different doses of CYT D. After 24 and 96 h (HUVEC) or 24 and 72 h (HDMEC) of treatment, 3-(4,5-Dimethylthiazol-2-yl)-2,5-diphenyltetrazolium bromide (MTT, 0.5 mg/ml; Sigma-Aldrich) was added in the culture medium in a ratio of 1:10. After 4 h of incubation with the MTT solution, formazan crystals, derived by the degradation of MTT into the mitochondria of living cells, were dissolved in ethanol/DMSO (1:1), and absorbance was measured at 550 nm using Varioskan LUX Multimode Microplate Reader (Thermo Fisher Scientific). Viability was calculated by comparing the absorbance of each treatment to the untreated (-) sample at 24 h. The experiments were repeated five times in triplicate.

For samples deriving from the RWV, the beads were collected from the vessels, washed with phosphate-buffered saline, trypsinized, stained with trypan blue solution (0.4%), and counted using Luna Automated Cell Counter (Logos Biosystems, Anyang, South Korea). The experiment was performed three times in triplicate.

### Western Blot

The cells were lysed in 10 mM Tris-HCl (pH 7.4) containing 3 mM MgCl_2_, 10 mM NaCl, 0.1% SDS, 0.1% Triton X-100, 0.5 mM EDTA, and protein inhibitors, and equal amounts of proteins were separated by SDS-PAGE on 4–20% Mini-PROTEAN TGX Stain-free Gels (Bio-Rad, Hercules, CA, United States) and transferred to nitrocellulose membranes by using Trans-Blot^®^ Turbo^TM^ Transfer Pack (Bio-Rad, Hercules, CA, United States). Antibodies against TRPM7 (Bethyl, Montgomery, TX, United States), heat shock protein HSP70 (Tebu Bio-Santa Cruz, Magenta, Italy), thioredoxin-interacting protein (TXNIP) (Invitrogen, Carlsbad, CA, United States), and HSP27 and phospho P-HSP27 (Cell Signaling, Euroclone, Pero, Italy) were used. Secondary antibodies labeled with horseradish peroxidase (Amersham Pharmacia Biotech Italia, Cologno Monzese, Italy) were used, and immunoreactive proteins were detected with Clarity^TM^ Western ECL substrate (Bio-Rad, Hercules, CA, United States). Images were captured with a ChemiDoc MP Imaging System (Bio-Rad, Hercules, CA, United States). Nitrocellulose sheets were used as control loading. The experiment was repeated at least four times, with similar results. A representative blot and the densitometric analysis performed using ImageJ are shown. Reciprocal variations between samples are calculated as fold change compared to the respective control (reference value = 1). More blots and relative quantification are available in the Supplementary Material.

### Confocal Imaging

HUVEC and HDMEC seeded on glass coverslip and treated or not with CYT D for 24 and 96 or 72 h, respectively, were fixed in phosphate-buffered saline containing 4% paraformaldehyde and 2% sucrose, pH 7.6, permeabilized with Triton 0.3%, and incubated with phalloidin-TRITC (Thermo Fisher Scientific) for 1 h at 4°C. For HDMEC on the beads, we utilized fluorescein isothiocyanate-labeled phalloidin (Sigma-Aldrich) as described (Carlsson et al., 2003). The nuclei were stained using DAPI (Thermo Fisher Scientific). Finally, the cells were mounted with ProLong^TM^ Gold Antifade Mountant (Invitrogen), and images were acquired using a 40× objective in oil by a LEICA SP8 confocal microscope. Images were analyzed using ImageJ.

### Reactive Oxygen Species Production

Reactive oxygen species (ROS) production was quantified using 2′-7′-dichlorofluorescein diacetate (DCFDA, Thermo Fisher Scientific) on HDMEC cultured in 1g condition or in simulated microgravity for 4 and 10 days. At the end of the experiment, the cells were rapidly transferred into black-bottomed 96-well plates (Greiner bio-one, Frickenhausen, Germany) and exposed for 30 min to 20 μM DCFDA solution. The emission at 529 nm of the DCFDA dye was monitored using Varioskan LUX Multimode Microplate Reader (Thermo Fisher Scientific). The results are the mean of three independent experiments performed in triplicate. Data are shown as the percentage of ROS levels in HDMEC cultured in the RWV vs 1g conditions (CTR) ± standard deviation.

### Statistical Analysis

Data are reported as means ± SD. The data of MTT assays were normally distributed, and they were analyzed using one-way repeated-measures ANOVA. The *p*-values deriving from multiple pairwise comparisons were corrected by the Bonferroni method. Statistical significance was defined for a *p*-value ≤0.05.

The data of western blots were analyzed using nonparametric *t*-test (Mann–Whitney). Densitometric analysis and the calculation of fold changes were performed and used for the statistical analysis. Regarding the figures, ^∗^*p* ≤ 0.05, ^∗∗^*p* ≤ 0.01, ^∗∗∗^*p* ≤ 0.001, and ^****^p ≤ 0.0001.

## Results

### Cytoskeletal Remodeling in 1g by CYT D in HUVEC and HDMEC

**FIGURE 1:**
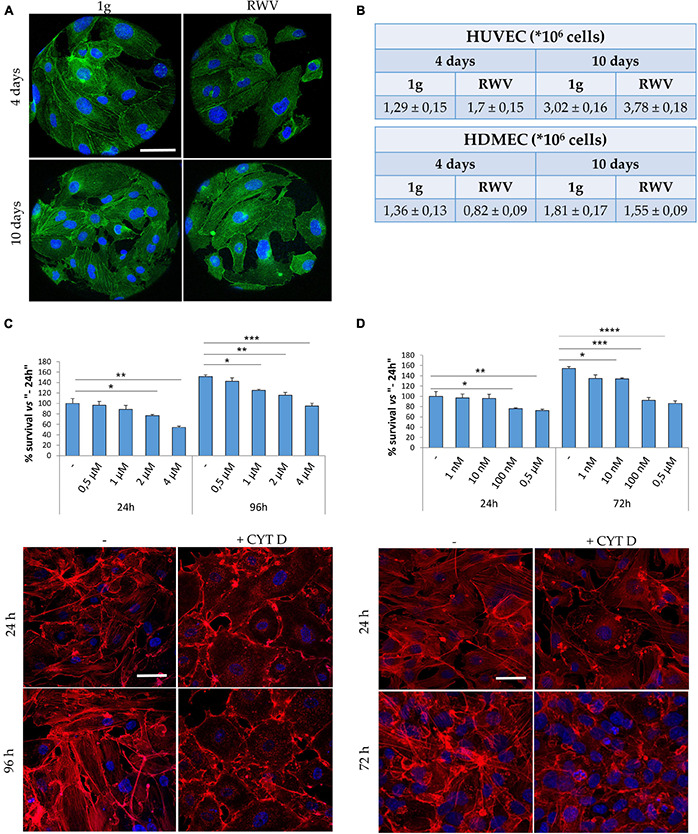
Cytoskeletal remodeling in cells cultured in simulated microgravity or exposed to CYT D. **(A)** Staining for actin (green) and nuclei (blue) in human dermal microvascular endothelial cell (HDMEC) grown on microcarrier beads in 1g or in the Rotating Wall Vessels (RWV) for 4 and 10 days. Scale bar: 40 μm. **(B)** Table showing the counts of human endothelial cell from umbilical vein (HUVEC) (upper table) and HDMEC (lower table) cultured in the RWV. **(C,D)** Different doses of CYT D were tested on HUVEC **(C)** and HDMEC **(D)**. MTT (upper panel) was performed after 24–96 or 24–72 h for HUVEC and HDMEC, respectively. Staining for actin (red) was performed at 24–96 h (HUVEC) or 24–72 h (HDMEC) in the presence of the selected dose (lower panels). Nuclear staining in blue. Scale bar: 40 μm. *P < 0.05, **P < 0.01, ***P < 0.001, and ****p < 0.0001.

Cytoskeletal remodeling is a common feature of eukaryotic cells exposed to real and simulated microgravity, including endothelial cells (Cotrupi et al., 2005; Versari et al., 2013; Barravecchia et al., 2018). In HUVEC grown in the RWV, we have shown that cytoskeletal disorganization plays a role in triggering mitophagy (Locatelli et al., 2020). Concerning HDMEC, Figure 1A shows cytoskeletal disruption and loss of stress fibers after 4 and 10 days of culture in the RWV (right panel) vs 1g (left panel). While simulated microgravity promotes cytoskeletal disruption in both HUVEC and HDMEC, the proliferative behavior of the cells is different since the growth of HUVEC was stimulated and the growth of HDMEC was retarded when cultured in the RWV (Figure 1B).

We then asked whether cytoskeletal alterations might govern, in part, endothelial response to gravitational unloading. To assess this issue, cells cultured in 1g were exposed to CYT D, a mycotoxin which depolymerizes the cytoskeleton and is largely utilized in experimental settings. It is noteworthy that CYT D is known to activate p53, thus determining growth arrest and apoptosis (Rubtsova et al., 1998). We treated HUVEC and HDMEC with different doses of CYT D for different times and then measured cell viability using the MTT assay which shows that CYT D is much more cytotoxic for HDMEC than for HUVEC (Figures 1C,D, upper panels). We selected 0.5 μM CYT D as the optimal concentration for HUVEC and 10 nM CYT D for HDMEC, doses which did not impair cell viability, for the following experiments. We then checked if these concentrations disrupted the cytoskeleton. HUVEC were treated with CYT D for 24–96 h and HDMEC for 24–72 h before staining with phalloidin-TRITC. In untreated HDMEC (Figure 1D, left panel), the actin fibers are short and thin, poorly organized, and with an enrichment of cortical actin once the cells reach confluence, while confluent untreated HUVEC (Figure 1C, left panel) possess a well-organized cytoskeleton, with thick stress fibers organized into bundles. CYT D effectively disorganizes the actin cytoskeleton in both HUVEC and HDMEC (Figures 1C,D, right panels).

### The Effect of Microgravity and Cytoskeletal Disruption in 1g on TRPM7 in HUVEC and HDMEC

**FIGURE 2:**
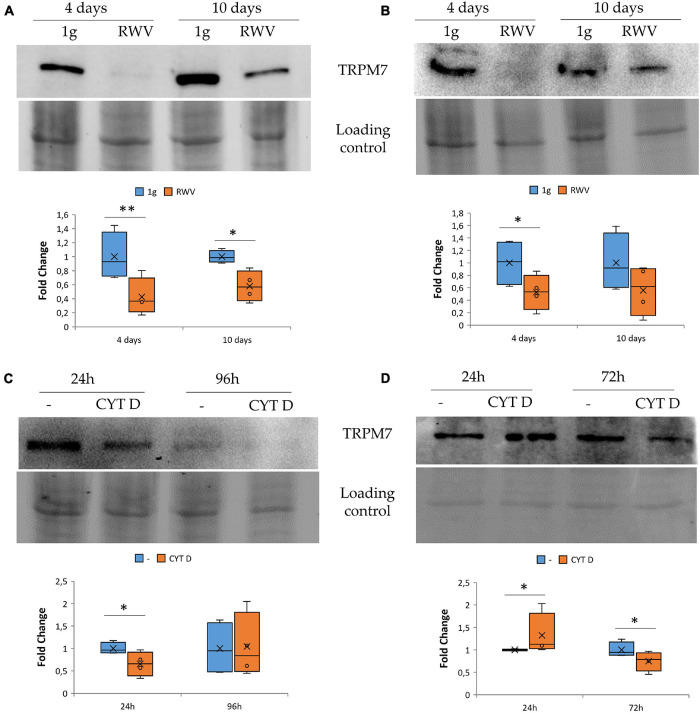
The effect of microgravity and cytoskeletal disruption in 1g on transient receptor potential melastatin 7 (TRPM7) in human endothelial cell from umbilical vein (HUVEC) and human dermal microvascular endothelial cell (HDMEC) the levels of TRPM7 were analyzed by western blot in HUVEC **(A,C)** and HDMEC **(B,D)** exposed to simulated microgravity [panels **(A,B)**, respectively] or to CYT D in 1g condition [panels **(C,D)** respectively]. Control loading was used to normalize, and the densitometric analysis is reported. *P < 0.05 and **P < 0.01.

We focused on TRPM7, a cation channel that contains an α-kinase domain (Nadler et al., 2001; Runnels et al., 2001). TRPM7 was reported to be a mechanosensor (Xiao et al., 2016; Negri et al., 2019) and also to be involved in the regulation of endothelial cell proliferation (Baldoli and Maier, 2012; Baldoli et al., 2013). HDMEC and HUVEC were maintained in the RWV for 4 and 10 days. This is the first report about the long-term effects of simulated microgravity in HDMEC. By western blot, we observed that HUVEC (Figure 2A) and HDMEC (Figure 2B) cultured in the RWV downregulate TRPM7 compared to their controls in 1g condition.

To assess whether cytoskeletal alterations play a role in modulating the levels of TRPM7, we cultured HUVEC and HDMEC in 1g in the presence of CYT D. We show that 24 h of treatment with CYT D downregulates TRPM7 in HUVEC (Figure 2C). Unexpectedly, in CYT D-treated HDMEC, TRPM7 is upregulated after 24 h and downregulated after 72 h (Figure 2D). These results indicate that the treatment with CYT D does not mimic what happens in HDMEC cultured in the RWV. More experiments are required to interpret the effect of CYT D in these cells at various time points.

### The Effect of Microgravity on Stress Response in HDMEC

**FIGURE 3:**
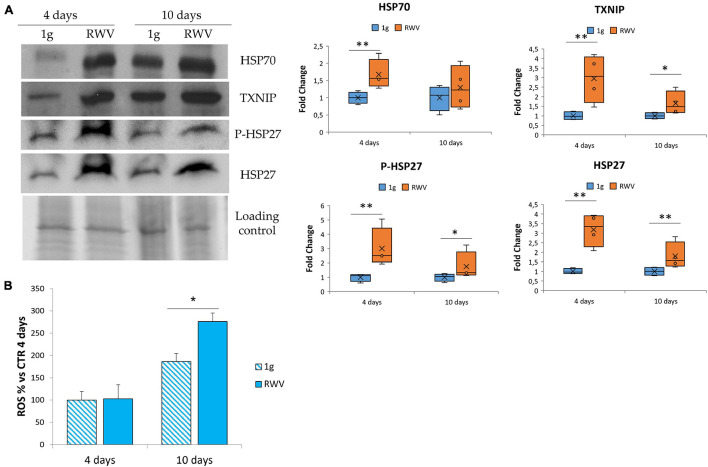
The effect of microgravity on stress response in human dermal microvascular endothelial cell (HDMEC). **(A)** Western blots performed on HDMEC cultured in microgravity for 4 and 10 days. Antibodies against TXNIP, HSP70, HSP27, and P-HSP27 were used and normalized on control loading. Densitometric analysis of the blot shown is reported on the right. **(B)** Reactive oxygen species production in HDMEC grown for 4 and 10 days in microgravity was analyzed using 2′-7′-dichlorofluorescein diacetate. The results were expressed as % compared to the sample grown in 1g condition for 4 days. *P < 0.05 and **P < 0.01.

In HUVEC, simulated microgravity induces the sequential involvement of various anti-oxidant proteins that ends up in counterbalancing the upregulation of pro-oxidant TXNIP so that no increase of ROS is detected (Cazzaniga et al., 2019). Since no data are available in HDMEC, we analyzed the total amounts of some stress proteins, i.e., HSP70, HSP27, P-HSP27, and TXNIP. Figure 3A shows the upregulation of HSP70, TXNIP, P-HSP27, and HSP27 in HDMEC grown in RWV compared to 1g controls. We also measured ROS accumulation in HDMEC grown in RWV and found an increase after 10 days in the RWV (Figure 3B).

### The Effect of Cytoskeletal Remodeling on Stress Response in HUVEC and HDMEC

We asked whether the modulation of stress proteins in simulated microgravity might be due to cytoskeletal remodeling. Indeed HSP70, HSP27, P-HSP27, and TXNIP were found to be linked to stress pathway as well as to cytoskeletal organization and actin stabilization (Quintá et al., 2011; Spindel et al., 2014; Sun et al., 2015). We analyzed their amounts after exposure to CYT D.

**FIGURE 4:**
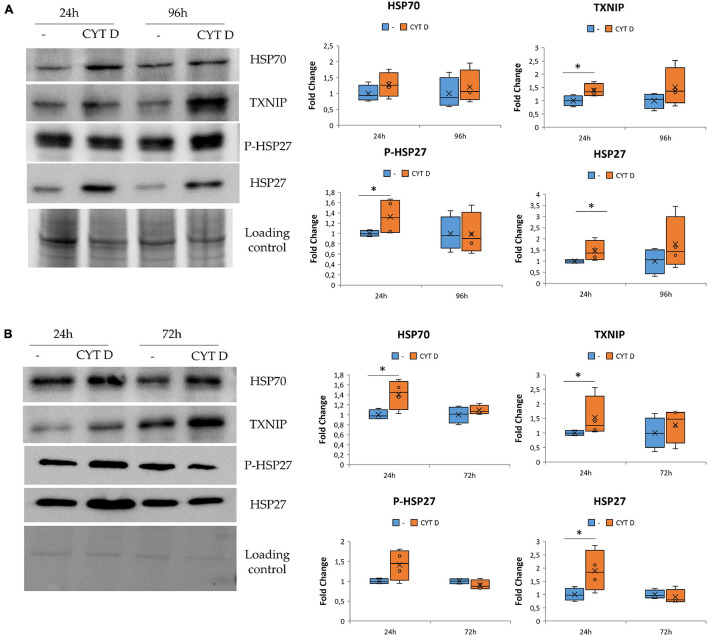
Cytoskeletal remodeling in the presence CYT D and the effect on stress response. Western blots were performed on human endothelial cell from umbilical vein **(A)** and human dermal microvascular endothelial cell **(B)**. Antibodies against TXNIP, HSP70, HSP27, and P-HSP27 were used and normalized on control loading. Densitometric analyses are shown next to the respective blots. *P < 0.05.

We cultured HUVEC for 24 and 96 h in control condition (−) or in the presence of 0.5 μM CYT D and then performed western blots on cell lysates (Figure 4A). We found that cytoskeletal disruption by 24 h exposure to CYT D was associated with the upregulation of TXNIP, HSP27, and its phosphorylated form. HSP70 was not significantly modulated by CYT D, differently from our previous observations in simulated microgravity (Carlsson et al., 2003; Cazzaniga et al., 2019). No differences were detected after 96 h of treatment with CYT D.

The same proteins involved in regulating stress response were studied also in HDMEC exposed to CYT D (Figure 4B). TXNIP, HSP70, and HSP27 were upregulated after 24 h of treatment with CYT D, whereas P-HSP27 is slightly but not significantly increased upon exposure to CYT D for 24 h. No differences were observed after exposure to CYT D for 72 h. In both HDMEC and HUVEC, it emerges that treatments with CYT D longer than 24 h do not simulate results obtained in the RWV, probably because CYT D has other effects on the cells beyond its action on the cytoskeletal network.

These results indicate that CYT D treatment does not completely mimic the stress response observed in EC cultured in microgravity (Figures 3A, 4B) but suggest the contribution of cytoskeletal alterations to orchestrating the stress response of HUVEC and HDMEC in microgravity.

## Discussion

The cytoskeleton is a highly dynamic network that structurally supports the cells. Moreover, it interacts with vesicles and organelles and controls their movement within the cell and is fundamental for endocytosis, migration, survival, and growth (Liu et al., 2015; Kast and Dominguez, 2017; Ohashi et al., 2017; Buracco et al., 2019; Hohmann and Dehghani, 2019; Moujaber and Stochaj, 2020). The cytoskeleton is sensitive to various stressful events and has been proposed as a sensor of changes of gravity (Vorselen et al., 2014). In EC, the content of actin filaments defines most of the mechanical properties (Janmaleki et al., 2016). Accordingly, shear stress reorganizes the actin network (Inglebert et al., 2020), and gravitational unloading rapidly shapes endothelial cytoskeleton and downregulates actin through transcriptional and post-transcriptional events (Carlsson et al., 2003; Cotrupi et al., 2005; Mariotti and Maier, 2008; Janmaleki et al., 2016). Among the components of endothelial cytoskeleton, actin filaments are the most sensitive to microgravity and respond with a dramatic drop of 65% compared to 26% reduction in microtubules (Janmaleki et al., 2016). Cytoskeletal disorganization is an event which occurs shortly after exposure to simulated microgravity and rapidly progresses thereafter. Indeed we have previously shown in HUVEC in the RWV that the disorganization of actin fibers begins after 4 h, and clusters of actin become evident in perinuclear position within 24 h. After 144 h in the RWV, these clusters disappear, and stress fibers are markedly decreased (Carlsson et al., 2003).

Because the cytoskeleton is continually remodeled to respond to different signals, several cytoskeleton-interacting proteins that stabilize it have been individuated. Among others, molecular chaperons interact closely with the cytoskeleton network and prevent undesired intermolecular interactions (Quintá et al., 2011). In particular, these proteins participate to the assembly/disassembly of cytoskeletal proteins and are key factors for many structural and functional rearrangements of actin during different physiological processes. HUVEC in simulated microgravity upregulate stress proteins (Cazzaniga et al., 2019), which play an important role in the adaptive response. Undeniably, the microvascular bed represents the majority of the total endothelial surface (Danese et al., 2007), and microvascular EC are the crucial players in inflammation (Danese et al., 2007) and angiogenesis (Carmeliet, 2005). Interestingly, an impairment of angiogenesis and wound healing in microgravity was reported (Kirchen et al., 1995; Unsworth and Lelkes, 1998; Davidson et al., 1999) and partially explained by the demonstration of cultured microvascular endothelial dysfunction in simulated microgravity (Cotrupi et al., 2005; Mariotti and Maier, 2008). For these reasons, we looked at stress proteins in HDMEC cultured in the RWV. These cells also sense microgravity as a stress and react by upregulating HSP70, HSP27, and TXNIP. HDMEC response seems to occur earlier than in HUVEC. HSP70 not only plays a role in maintaining cell viability but also contributes to the correct folding of cytoskeletal proteins (Quintá et al., 2011). In HUVEC, we have shown that HSP70 is important in the early adaptation to microgravity since it protects them against cell death (Cazzaniga et al., 2019). We hypothesize a similar function for HSP70 in HDMEC since no signs of apoptosis were detected in gravitationally unloaded cells (Mariotti and Maier, 2008). HSP27 is an anti-oxidant because it increases the amounts of glutathione and also prevents apoptosis (Katsogiannou et al., 2014). The antioxidant activity of HSP27, however, does not suffice to counterbalance the upregulation of the pro-oxidant TXNIP. Indeed differently from HUVEC (Cazzaniga et al., 2019), HDMEC in simulated microgravity accumulate more ROS than controls in 1g, and we hypothesize that oxidative stress might play a role in retarding their proliferation. Under stress, HSP27 can be reversibly phosphorylated. P-HSP27 regulates actin filament dynamics in cytoskeleton organization (Katsogiannou et al., 2014). This function might be important in microgravity to safeguard the newly remodeled cytoskeleton. As for TXNIP, it was demonstrated to be overexpressed in HUVEC after 10 days onboard the ISS (Versari et al., 2013) and upregulated in HUVEC in the RWV (Cazzaniga et al., 2019). While in HUVEC TXNIP was upregulated after 10 days, in HDMEC, it increases earlier, i.e., after 4 days of culture in the RWV. In EC, TXNIP activates Src, and Src activation correlates with increased F-actin stress fiber formation (Spindel et al., 2014). Therefore, it is feasible to propose that the increase of TXNIP might represent a mechanism to restrain cytoskeletal alterations. Moreover, since TXNIP stimulates the expression of VEGF receptor 2 in EC (Park et al., 2013), TXNIP upregulation might play a role in potentiating endothelial survival under stress.

Recently, we have shown in HUVEC that the disruption of the cytoskeleton by exposure to CYT D closely resembles microgravity-induced cytoskeletal disorganization and mitochondrial dynamics (Locatelli et al., 2020). Accordingly, disrupting the cytoskeleton with CYT D in HUVEC mimics the effect of simulated microgravity on the levels of HSP27 and TXNIP but not of HSP70. We hypothesize that other mechanisms, which need to be disclosed with more experiments, are involved in increasing HSP70 in HUVEC grown in the RWV. In HDMEC, CYT D mimics the microgravity-dependent upregulation of HSP70, HSP27, and TXNIP. Differently from results obtained in simulated microgravity, no significant differences of P-HSP27 were detected in CYT D-treated HDMEC. We hypothesize that CYT D might prevent the phosphorylation of HSP27 as previously shown in chick mesenchymal stem cells where CYT D impaired the phosphorylation of cofilin (Kim et al., 2012). Alternatively, CYT D-induced actin disorganization might disrupt the association of actin with various regulatory proteins, among which are kinases and phosphatases, thereby interfering with their activity or with their capability to target specific substrates (Elizarov and Vassetzky, 2003).

In general, our results suggest that it is actin disorganization that prompts most of the stress responses activated by these cells so that they can cope with the reduction of gravitational forces.

We also demonstrate the downregulation of TRPM7 in HUVEC grown in simulated microgravity or after exposure to CYT D. Since TRPM7 is a cation channel that has been shown to serve as a mechanosensor (Negri et al., 2019), we interpret its reduction as an adaptive response to a decrease of mechanical forces on the cells. It is noteworthy that silencing TRPM7 accelerates HUVEC growth, partly through the erk signaling pathway (Inoue and Xiong, 2009; Baldoli et al., 2013). Therefore, decreased amounts of TRPM7 might account for HUVEC growth stimulation in simulated microgravity (Carlsson et al., 2003; Versari et al., 2007). Like HUVEC, HDMEC show reduced amounts of TRPM7 when cultured in the RWV. It is of note that silencing TRPM7 as well as simulated microgravity retard HDMEC growth (Mariotti and Maier, 2008; Baldoli and Maier, 2012). We anticipate that decreased amounts of TRPM7 might be implicated in inhibiting HDMEC growth in simulated microgravity. We therefore propose that alterations of the cytoskeleton only partially mediate the effects of gravitational unloading on TRPM7 levels since CYT D downregulates TRPM7 only at one time point.

In conclusion, some differences emerge between HUVEC and HDMEC in the kinetics of the response to gravitational unloading and in the accumulation of ROS. This is not unexpected due to the fact that the endothelium is highly heterogeneous. Our studies might have implications also in other contexts. Indeed simulating microgravity is becoming a useful tool to unveil the role of mechanical stimuli in health and disease (Poon, 2020) and to optimize protocols for tissue engineering and regenerative medicine (Grimm et al., 2018), thus answering the questions raised by Rinaldi (2016) about the meaning of space research.

## Data Availability Statement

The original contributions presented in the study are included in the article/Supplementary Material, further inquiries can be directed to the corresponding author.

## Author Contributions

LL and JM contributed to conceptualization, formal analysis, and writing—review and editing. LL contributed to the methodology and investigation. JM contributed to the writing—original draft preparation, and funding acquisition. Both authors have read and agreed to the published version of the manuscript.

## Conflict of Interest

The authors declare that the research was conducted in the absence of any commercial or financial relationships that could be construed as a potential conflict of interest.

## Publisher’s Note

All claims expressed in this article are solely those of the authors and do not necessarily represent those of their affiliated organizations, or those of the publisher, the editors and the reviewers. Any product that may be evaluated in this article, or claim that may be made by its manufacturer, is not guaranteed or endorsed by the publisher.
